# A preliminary study exploring the change in ankle joint laxity and general joint laxity during the menstrual cycle in cis women

**DOI:** 10.1186/s13047-021-00459-7

**Published:** 2021-03-24

**Authors:** Tomomi Yamazaki, Sae Maruyama, Yuki Sato, Yukako Suzuki, Sohei Shimizu, Fumiya Kaneko, Masahiro Ikezu, Kanta Matsuzawa, Mutsuaki Edama

**Affiliations:** Institute for Human Movement and Medical Sciences, Niigata University of Health and Welfare, 950-3198, Shimami-cho 1398, Kita-Ku, Niigata, Japan

**Keywords:** Anterior tibiofibular ligament, Ovulation phase, Lateral ankle ligamentous sprain

## Abstract

**Background:**

The purpose of the present study was to examine the relationship between ankle joint laxity and general joint laxity (GJL) in relation to the menstrual cycle, which was divided into four phases based on basal body temperature and ovulation, assessed using an ovulation kit.

**Methods:**

Participants were 14 female college students (21–22 years) with normal menstrual cycles (cis gender). Anterior drawer stress to a magnitude of 120 N was applied for all participants. Anterior talofibular ligament (ATFL) length was measured as the linear distance (mm) between its points of attachment on the lateral malleolus and talus using ultrasonography. Data on ATFL length from each subject were used to calculate each subject’s normalized length change with anterior drawer stress (AD%). The University of Tokyo method was used for evaluation of GJL. AD% and GJL were measured once in each menstrual phase.

**Results:**

There was no statistically significant difference between AD% in each phase. GJL score was significantly higher in the ovulation and luteal phases compared with the early follicular phase. AD% and GJL showed a positive correlation with each other in the ovulation phase.

**Conclusions:**

Although it is unclear whether estrogen receptors are present in the ATFL, the present study suggests that women with high GJL scores might be more sensitive to the effects of estrogen, resulting in ATFL length change in the ovulation phase.

## Introduction

It was previously reported that the frequency of sports injuries in women is higher than that in men, suggesting a relationship between the menstrual cycle and sports injury [1, 2]. The menstrual cycle is controlled mainly by cyclic fluctuations in estradiol and progesterone [3], and is classified primarily into follicular, ovulation and luteal phases.

Several studies [3–7] investigating the timing of injury of the anterior cruciate ligament (ACL) of the knee in relation to the menstrual cycle reported that ACL injuries often occur during the follicular [3, 5] and ovulation phases [4, 6]. It has also been reported that estrogen receptors are present in the human ACL [8], and that female hormones affect the tissue structure of the ACL [9]. In vivo studies have reported that anterior knee laxity [10] increases during ovulation [11] and luteal phases [12]. Additionally, plantar fasciitis, a type of sports injury, is more common in women. Previous studies have investigated female hormone levels in relation to plantar fascia elasticity, and reported that plantar fascia elasticity increases during ovulation, when estrogen levels are at their peak [13]. Thus, changes in the elasticity and joint laxity of ligaments and tendons have been observed in each phase of the menstrual cycle, and their relationship with sports injuries has been discussed.

Lateral ankle ligamentous sprain (LAS) is one of the most common injuries resulting from recreational activities and competitive sports [14]. Of them, roughly 66–85 % involve injuries to the anterior talofibular ligament (ATFL) alone [15–17]. The intrinsic predictive factors of LAS include anatomic characteristics, functional deficits in isokinetic strength, flexibility, joint position sense, muscle reaction time, postural stability, gait mechanics, limb dominance, previous ankle sprains, and body mass index [14]. In recent years, generalized joint laxity (GJL) has also been shown to be a risk factor for ACL injury [18]. Stettler et al. [19] reported higher values for AKL in individuals with higher GJL scores compared to those with normal mobility. In addition, GJL scores are higher in women than in men [20]; this difference between men and women has been attributed to differences in sex hormone levels. LASs have also been reported to occur more frequently in women than in men [10]. However, the effects of hormone fluctuations in women on ankle joint laxity and GJL have not been investigated.

The previous studies have described the usefulness of ultrasonography in diagnosing ankle ligament injuries [21, 22]. The application of anterior drawer stress during ultrasonography examination has allowed the evaluation of the changes in location between the ATFL origin and insertion [21]. The use of ultrasonography has demonstrated good-to excellent interrater reliability in the linear measurement of the ATFL under stress positions using the Telos stress device [22, 23]. Therefore, in this study, ankle joint laxity is measured using ATFL ratio of stress ultrasonography.

The purpose of the present study was to examine the relationship between ankle joint laxity and GJL during the menstrual cycle, divided into four phases based on basal body temperature (BBT) and ovulation, assessed using an ovulation kit. We hypothesized that ankle joint laxity and GJL values were high during the ovulation period when estrogen levels are high.

## Methods

### Participants

We surveyed 49 female university students (cis gender) using a questionnaire and interview. Inclusion criteria were as follows: (1) no history of varus and valgus sprains in the past 6 months; (2) no history of surgery on the lower leg; (3) no oral contraceptive or other hormone-stimulating medication usage in the preceding 6 months [12]; and (4) physically active less than three times per week. Among the students who were screened, 14 women (mean age, 21.1 ± 0.3 years; mean height, 159.0 ± 4.5 cm; mean weight, 53.0 ± 6.1 kg; mean cycle days, 30.1 ± 2.8 days) with regular menstrual cycles and biphasic BBTs (indicative of ovulatory cycles) were enrolled. This study was approved by the University Ethics Review Committee (Approval Number 17,946). In addition, this study complied with the Declaration of Helsinki, and was conducted only after written consent was obtained from the study participants, who had been fully informed (in both verbal and written form) of the nature of the experiment.

### Evaluation of the menstrual cycle

**Fig. 1:**
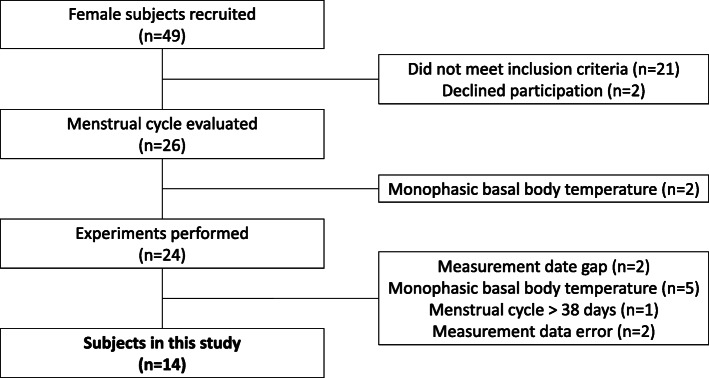
Subject selection

 Based on the completed questionnaires and interviews conducted in the 49 female subjects, we asked 26 of them who had regular menstrual cycles and agreed to participate in this study to measure and record their BBT every morning for 1 to 2 months preceding the start of the experiment. Subjects were provided with basal thermometers (Citizen Electronic Thermometer CTEB503L, Citizen Systems Co., Ltd., Tokyo, Japan) for this purpose. To estimate the ovulation date, subjects were provided with ovulation kits (Doctor’s Choice One Step Ovulation Test Clear; Beauty and Health Research, Inc., CA, USA) to be used from the day after the end of menstruation. Since luteinizing hormone (LH) in urine and serum have been shown to correlate with each other [24], the ovulation date was estimated using the ovulation kit results as a substitute for blood sampling. A recording sheet for creation of a BBT table was prepared, and daily BBT, menstrual period, and ovulation kit results were recorded. Based on these data, the first day of menstruation was considered day 1, and the mean BBT up to day 6 was calculated. When the BBT for three consecutive days after ovulation (as determined by the ovulation kit) was at least 0.2 °C higher than this mean value, it was judged that the subject exhibited a biphasic cycle of low and high temperatures [25]. Women with biphasic cycles were classified as having a normal ovulatory pattern, while those with monophasic cycles were considered to have an anovulatory pattern [25, 26]. Of the 26 women whose menstrual cycles were monitored, two were excluded because their BBT was monophasic; ATFL and GJL were measured in the remaining 24 subjects. The final enrolled study population consisted of 14 women who had a cycle length of 25 to 38 days [27] and biphasic BBTs during the menstrual cycle, and in whom ATFL length and GJL measurements were performed. Ten of the 24 subjects were excluded for the reasons indicated in Fig. 1.

### Timing of measurement

ATFL length and GJL measurements were taken once in each of the four phases of the menstrual cycle; these phases consisted of the early follicular phase, late follicular phase, ovulatory phase, and luteal phase.

ATFL and GJL were measured in the early follicular phase from 3 to 4 days after the start of menstruation, in the late follicular phase from 3 to 4 days after the end of menstruation, in the ovulation phase from 2 to 4 days after the day when the ovulation kit indicated a positive result, and in the luteal phase from 5 to 10 days after the start of the high temperature phase. In consideration of possible diurnal variations, all measurements in all subjects were performed between 8:00 a.m. and 12:00 p.m.[28].

### Measurement methods

**Fig. 2:**
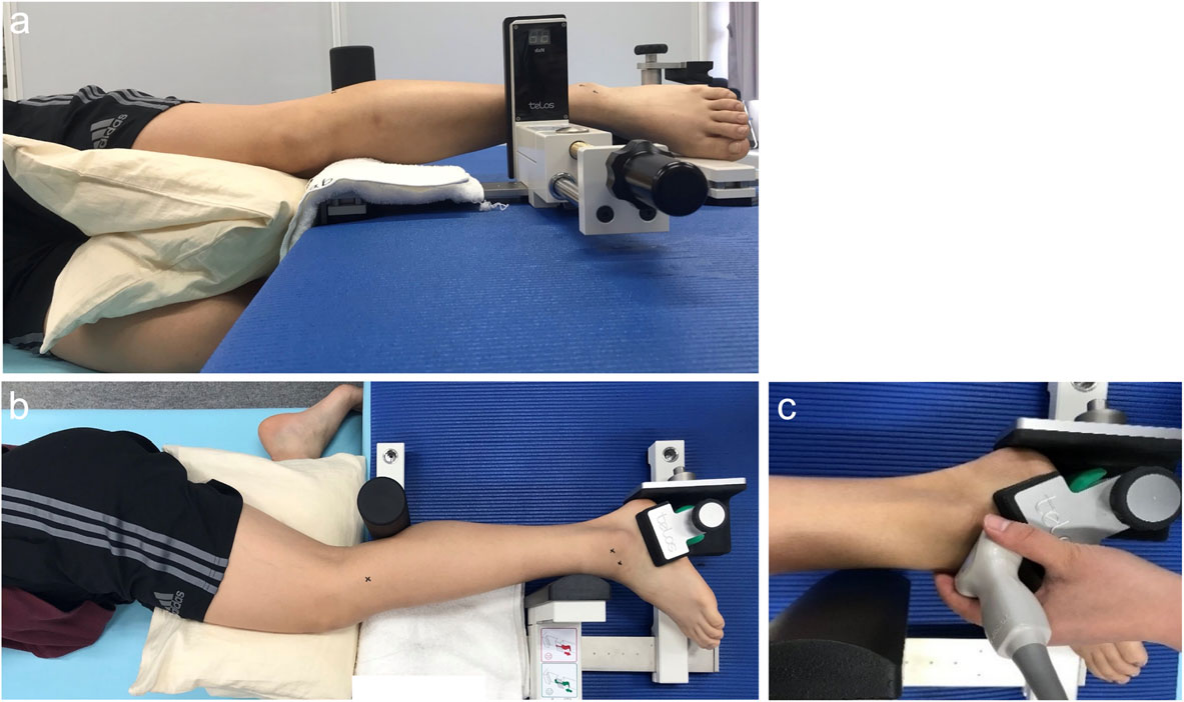
Position of the foot during measurement Neutral ankle position with about 30° of plantar flexion, with the subject lying on their side and the lower extremity placed on the bed. Ankle stress conditions were applied with a Telos Stress Device. Anterior drawer stress was applied using a force of magnitude 120 N for all subjects. **a**: Side view. **b **front view. **c **probe position

**Fig. 3:**
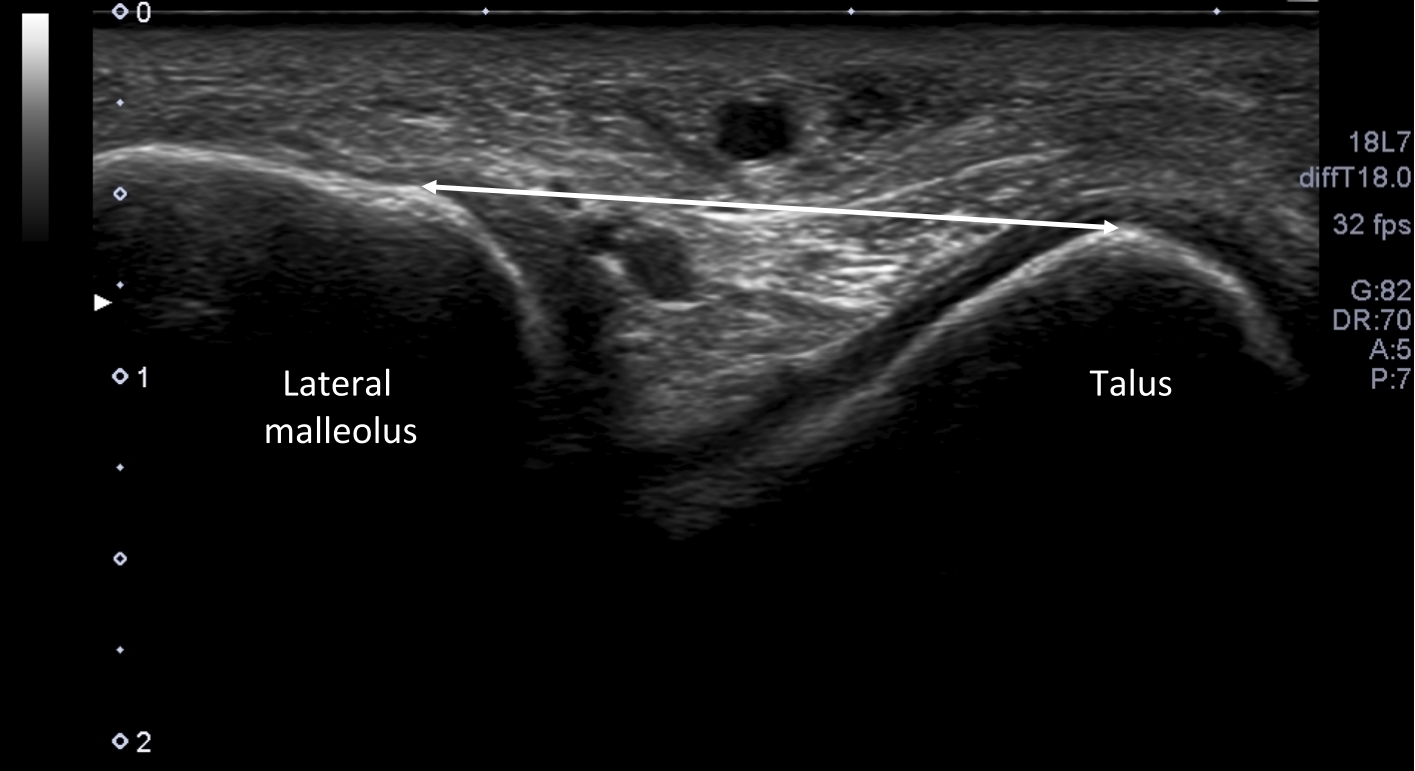
Ultrasound image for measurement of the anterior talofibular ligament length. The ultrasound image captured directly over the anterior talofibular ligament origin and insertion allows the examiner to use a straight line measurement tool to draw a line from the anterolateral aspect of the lateral malleolus to the talus, points that correspond to the anatomic attachment sites of the ligament

Ultrasound imaging was performed using ultrasonography (Aplio 500, Toshiba Medical Systems, Tochigi, Japan) with a 10-MHz linear probe. The test positions used were identical to those in a previous study [23, 29] and were performed in the following order: (1) neutral ankle position with about 30° of plantar flexion, with the subject lying on their side and the lower extremity positioned on the bed; and (2) anterior drawer stress to the ankle, performed about 3 cm proximal to the lateral malleolus (Fig. 2). Ankle stress conditions were applied with a Telos Stress Device (Telos SE, Aimedic MMT, Japan). Anterior drawer stress was applied to a magnitude of 120 N for all participants. The measurement was performed thrice, once each by three examiners, two examiners performing the test using ultrasonography, and one examiner performing the test using the Telos Stress Device. With the participant’s ankle in approximately a neutral position, the examiner palpated the anterolateral aspect of the lateral malleolus and talus. Next, the examiner applied ultrasound conducting gel over the lateral aspect of the ankle and positioned the ultrasound probe. The examiner then oriented the probe to view the cross-sectional representation of the lateral malleolus, kept on the right side of the screen, while the lateral talar articular surface cartilage and the neck of the talus, where the ATFL attaches, were identified (Fig. 3). After optimizing the image and centering these bony landmarks within the field of view, the examiner saved the three images and removed the probe. Next, the stress device was applied to the ankle and three images of the ATFL were obtained while performing the anterior drawer stress by application of a posteriorly directed force of 120 N through the tibia (Fig. 2).

Ultrasonographic image analysis was performed using an ultrasonic diagnostic imaging system. The ATFL length was measured as the linear distance (mm) between the landmarks. The anterolateral aspect of the lateral malleolus was identified as the ATFL origin, and the peak of the talus was used as the insertion point. The average of the values measured from the three images was adopted. ATFL length data from each subject were.

used to calculate each participant’s normalized length change with application of anterior drawer stress (AD%) using the formula [(L stress – L neutral) /L neutral] ×100, where L is the ATFL length in millimeters [29].

**Fig. 4:**
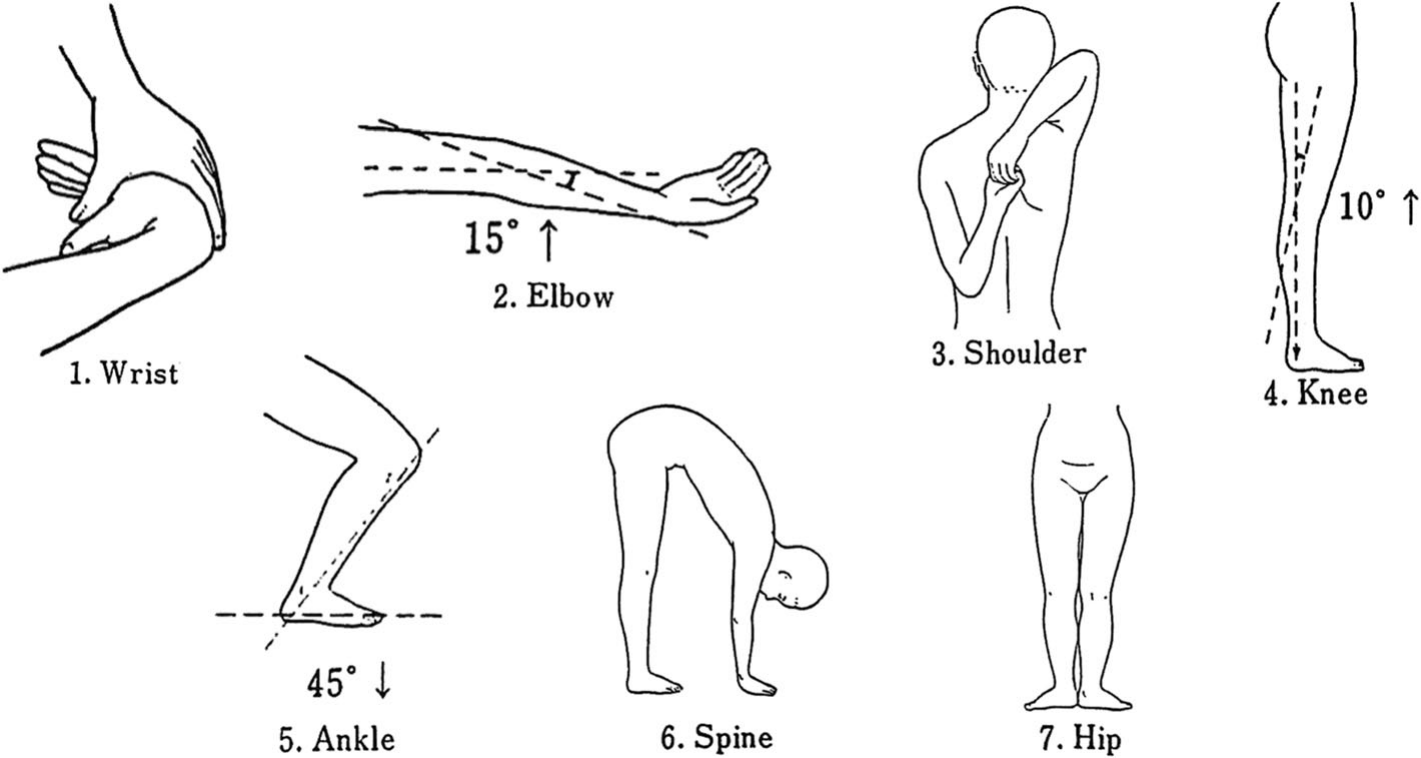
The University of Tokyo joint laxity test. Laxity of six major joints in the body (hip, knee, ankle, shoulder, elbow, wrist) and of the spine were examined. Each item was assigned a value of 1 point (0.5 points each on the left and right sides for bilateral joints) , for a total of 7 points

GJL was measured using the University of Tokyo joint laxity test [30] (Fig. 4). Mobility was measured at the spine, and bilaterally at the hip, knee, ankle, shoulder, elbow and wrist. Each item was assigned a value of 1 point, and a total of seven positions were measured; for the six major bilateral joints (i.e., aside from the spine), the left and right positions were assigned a value of 0.5 points each. For items with joint angle as the criterion, the joint angle was measured using a goniometer. Joint angle measurements were performed by one operator and recorded by one operator.

### Intra‐rater reliability

To assess the inter-rater reliability of the measurements, 10 adult men (mean age, 21.0 years; mean height, 176 ± 6.5 cm; mean weight, 68.9 ± 6.3 kg) were subjected. The study content was fully explained to the participants, and written, informed consent was obtained from all participants. Measurement was performed using the above-described ATFL length measurement method; again, the measurement was performed three times, and the average of the three measurements was used. The measurement was repeated on two or more separate days within 1 week, and the intraclass correlation coefficient (ICC) (1, 3) was calculated. The resulting ICC (1, 3) for the ATFL measurements was 0.92 (neutral ATFL length) and 0.93 (stress ATFL length) (Table 1). According to the criteria of Landis et al., [31] reproducibility is considered to be almost perfect when the ICC is 0.81 or more. Therefore, the reproducibility of ATFL length measurement in this study was considered to be high.

**Table 1:** Reliability of ultrasonography measurements

Load	ATFL length (mm)		ICC (1,3)	Reliability	MDD_95 %_
	**First Rater**	**Second Rater**			
Neutral	21.9 ± 2.4	21.7 ± 2.5	0.929	almost perfect	1.8
120 N stress	23.2 ± 2.7	23.1 ± 2.2	0.920	almost perfect	1.9

*n* = 10

Values are given as mean ± SD

*ATFL* anterior talofibular ligament; *ICC* intraclass correlation coefficient

MDD95 %: minimal detectable difference at the 95 % confidence interval

### Statistical analysis

Statistical analyses were performed using SPSS (version 24.0, SPSS Japan Inc., Tokyo, Japan). A one-way repeated measures analysis of variance was used to compare AD% and GJL in each phase of the menstrual cycle. Pearson’s correlation test was used to assess the relationship between AD% and GJL in each phase. Pearson’s chi-squared test was used to compare differences in assessments at the spine, and bilaterally at the hip, knee, ankle, shoulder, elbow and wrist in the ovulation phase. The level of significance was set at 5 %.

## Results

**Fig. 5:**
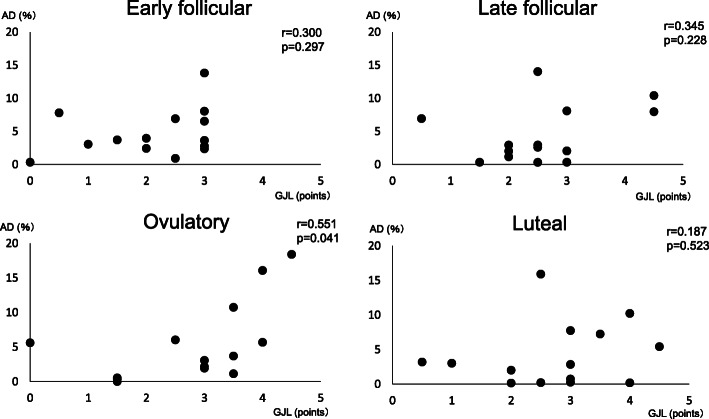
Correlation between general joint laxity and anterior talofibular ligament length change with anterior drawer stress in each cycle AD (%): anterior talofibular ligament length change with anterior drawer stress GJL: general joint laxity

There was no statistically significant difference between AD% in each phase (Table 2). GJL score was significantly higher in the ovulation (*p* = 0.016) and luteal phases (*p* = 0.026) compared with the early follicular phase (Table 3). AD% and GJL showed a positive correlation only in the ovulation phase (*R* = 0.551, *P* = 0.041) (Fig. 5). In all phases, there were a statistically significant number of ankle (*p* = 0.001) and shoulder (*p* = 0.001) joints that were positive for GJL (Table 4).

**Table 2:** Change in anterior talofibular ligament length with the anterior drawer test (%) during the menstrual cycle

	Early follicular phase	Late follicular phase	Ovulation phase	Luteal phase
ATFL length change (%)	4.7 ± 3.6	4.4 ± 4.3	5.6 ± 5.7	4.0 ± 4.8

*n* = 14

Values represent means ± SD

*ATFL* anterior talofibular ligament

There was no statistically significant difference between AD% in each phase

**Table 3:** Change in general joint laxity during the menstrual cycle

	Early follicular phase	Late follicular phase	Ovulation phase	Luteal phase
General joint laxity				
Score (points)	2.1 ± 1.0	2.6 ± 1.1	2.8 ± 1.3*	2.8 ± 1.1**

*n* = 14

Values represent means ± SD

*P = 0.016 vs. early follicular phase

**P = 0.026 vs. early follicular phase

**Table 4:** Number of subjects positive for general joint laxity during the menstrual cycle

	Spine	Hip	Knee	Ankle	Shoulder	Elbow	Wrist
**Early follicular**	3/11	0/14**	5/9	9/5**	8/6**	1/13**	4/10
**Late follicular**	5/9	0/14*	4/10	9/5*	9/5*	1/13*	4/10
**Ovulation phase**	6/8	0/14*	4/10	10/4*	9/5*	2/12*	6/8
**Luteal phase**	6/8	1/13**	3/11	11/3**	9/5**	0/14**	5/9

*n* = 14

Data represent the Number of Positives (N) / Number of Negatives (N)

*P = 0.001 vs. Negatives

**P < 0.001 vs. Negatives

## Discussion

In this study, there was no statistically significant difference between AD% during the four phases of the menstrual cycle. GJL score, however, was significantly higher in the ovulation and luteal phases as compared with the early follicular phase. Further, AD% and GJL showed a positive correlation in the ovulation phase. Regarding the relationship between the menstrual cycle and tissue structure of the ACL, it has been reported that estrogen receptors are present in the human ACL [8], and in vivo studies have reported that AKL increases during ovulation [11] and luteal phases [12]. Higher values for AKL in individuals with higher GJL scores compared to those with normal mobility have also been reported [19]. Previous studies have investigated the correlation between female hormones and plantar fascia elasticity, and reported that plantar fascia elasticity increases during ovulation, synchronous with high estrogen levels [13]. Therefore, although it is unclear whether estrogen receptors are present in the ATFL, it was suggested that women with high GJL scores might be more sensitive to the effects of estrogen on ATFL length change in the ovulation phase. Also, this study was considered to be one of the causes of LAS occurring in women.

In our study, there was a large standard deviation between individuals. In a previous study, patients were divided into three groups according to LAS severity for comparison of the ATFL elongation rate. The results showed that ATFL length change with the anterior drawer test in the control group was 1.3 ± 10.7 %, in the history of 1 ankle sprain more than 1 year ago and no residual symptoms of instability or giving way (Coper) group was 14.0 ± 15.9 %, and that in the chronic ankle instability (CAI) group was 15.6 ± 15.1 %, indicating significantly higher ATFL length change in the Coper and CAI groups as compared to the control group [29]. Due to the inclusion criterion of “no history of varus and valgus sprains in the past 6 months” used in our study, it is possible that our study included subjects in both the Coper and CAI groups. Future studies should consider the subjects’ past history, including the severity of ankle sprain. In addition, this study is small sample sizes. This may cause a large standard deviation between individuals of this study.

Several limitations must be considered in this study. First, we did not measure hormone levels to clearly differentiate the four phases of the menstrual cycle. Instead of measuring hormone concentrations by blood sampling, we performed cycle classification using an ovulation kit, which is an inexpensive and non-invasive tool, and the BBT method. Since a correlation has been shown between urinary and serum luteinizing hormone levels, we inferred that the ovulation phase could be adequately defined using an ovulation kit. In addition, use of the BBT method enables estimation of ovulatory and anovulatory cycles [25, 26]. Thus, we expected that including subjects whose BBT showed a biphasic trend would enable us to select subjects with normal ovulatory cycles whose menstrual cycle could be classified into four phases. However, since the timing and phasing of estrogen and progesterone concentration changes vary considerably across the menstrual cycle [28, 32, 33], it might be necessary to classify menstrual cycle according to hormone concentrations in serum, urine, or saliva in future studies. The second limitation is the sample size. The total number of subjects in this study was 14. This may limit the interpretation of the results of this study and therefore requires a larger sample size. The third limitation is due to the inclusion criterion of “no history of varus and valgus sprains in the past 6 months” used in our study, it is possible that our study included subjects in both the Coper and CAI groups.

## Conclusions

It is unclear whether estrogen receptors are present in the ATFL, although it has been suggested that women with high GJL scores might be more sensitive to the effects of estrogen on ATFL length change during the ovulation phase. Also, this study was considered to be one of the causes of LAS occurring in women. In future studies, menstrual cycle phases should be identified by measuring hormone concentrations in order to fully examine the effects of the menstrual cycle on the risk factors of LAS.

## Data Availability

The data that support the findings of this study are available from the corresponding author upon reasonable request.
